# Effective Pollen-Fertility Restoration Is the Basis of Hybrid Rye Production and Ergot Mitigation

**DOI:** 10.3390/plants11091115

**Published:** 2022-04-20

**Authors:** Thomas Miedaner, Viktor Korzun, Peer Wilde

**Affiliations:** 1 State Plant Breeding Institute, University of Hohenheim, Fruwirthstr. 21, 70799 Stuttgart, Germany; 2 KWS SAAT SE & Co. KGaA, Grimsehlstr. 31, 37555 Einbeck, Germany; viktor.korzun@kws.com; 3 KWS LOCHOW GMBH, Ferdinand-von-Lochow-Str. 5, 29303 Bergen, Germany; peer.wilde@kws.com

**Keywords:** *Claviceps purpurea*, CMS, hybrid breeding, pollen, restorer genes, yield

## Abstract

Hybrid rye breeding leads to considerably higher grain yield and a higher revenue to the farmer. The basis of hybrid seed production is the CMS-inducing Pampa (P) cytoplasm derived from an Argentinean landrace and restorer-to-fertility (*Rf*) genes. European sources show an oligogenic inheritance, with major and minor *Rf* genes, and mostly result in low-to-moderate pollen-fertility levels. This results in higher susceptibility to ergot (*Claviceps purpurea*) because rye pollen and ergot spores are in strong competition for the unfertilized stigma. *Rf* genes from non-adapted Iranian primitive rye and old Argentinean cultivars proved to be most effective. The major *Rf* gene in these sources was localized on chromosome 4RL, which is also a hotspot of restoration in other *Triticeae*. Marker-based introgression into elite rye materials led to a yield penalty and taller progenies. The *Rfp1* gene of IRAN IX was fine-mapped, and two linked genes of equal effects were detected. Commercial hybrids with this gene showed a similar low ergot infection when compared with population cultivars. The task of the future is to co-adapt these exotic *Rfp* genes to European elite gene pools by genomic-assisted breeding.

## 1. Rye Pollination and Ergot, a Story of Competition

Rye (*Secale cereale* L.) originates from an area extending from central and eastern Turkey through nortwesten Iran to Transcaucasia. It was probably not cultivated continuously in its area of origin. When rye came to northern Europe as a weed, it was able to exploit its advantages of superior winter hardiness and modest nutrient requirements and became the dominant cereal from the early Middle Ages [1]. Initially, rye was grown in a vast region from northern France to eastern Siberia. Nowadays, rye-growing is still centered in Europe, where 86% of grains are produced, with Germany, Poland, the Russian Federation, Denmark, and Belarus as the main producers in 2019 [2]. In rye as a cross-pollinating crop, population and hybrid cultivars are available. Hybrid breeding started in the 1970s at the University of Hohenheim, Germany, and in 1985 the world’s first three hybrid cultivars were released [3]. This breeding method revolutionized rye-growing, because hybrid breeding provided about 20% higher grain yield and a threefold higher gain from selection, compared to population rye, over a 30-year period [4]. Nowadays, about 80% of the German rye-growing area is planted with hybrid rye cultivars [5], and hybrid rye is available in all other main production countries, as well as in Austria, the USA, and Canada.

**Figure 1:**
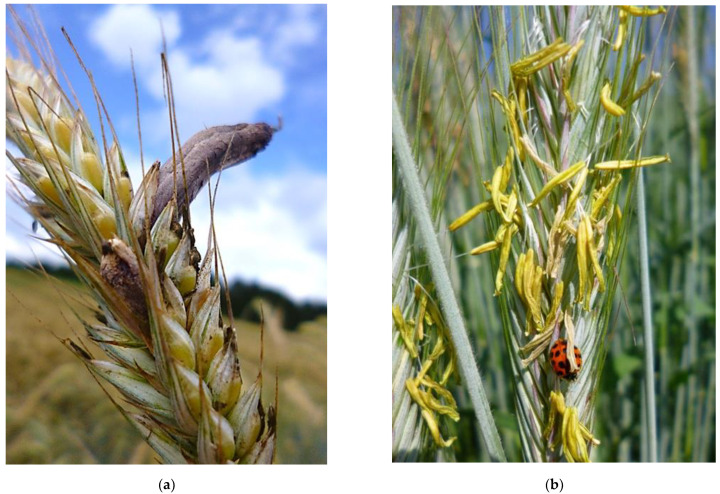
(**a**) Ergot sclerotia in a ripening rye head; (**b**) Full pollen-fertile rye during flowering.

Since Medieval times, the results of ergot infections have been documented. Rye and ergot were so closely interwoven that botanical drawings of rye in the 17th century included ergot. Ergot refers to the blackish purple sclerotia of the fungus *Claviceps purpurea* (Fries ex Fries) Tulasne that extend far beyond the husks of rye (Figure 1a). They contain more than 80 ergot alkaloids, of which six alkaloids and their corresponding epimers were found to be the most important ones in Europe. Prolonged consumption of ergot-containing flour, which was common among the peasant population in the Middle Ages, leads to a serious disease, ergotism, that is characterized either by nervous disorders (fever, muscle spasms, tremor, paralysis, hallucinations) or by vasoconstriction effects (gangrene, violent burning, shooting pain in the fingers and toes, and “St. Anthony’s fire”). All mammalian livestock are affected by ergot contamination of feed [6]. Epidemic outbreaks in the Middle Ages resulted in a death rate of between 10% and 20% and a reduction in the birth rate due to the premature onset of labor and miscarriages [7]. The main factor for ergot epidemics is rainy weather during rye flowering. High humidity stimulates fungal growth and reduces the availability of rye pollen. The fungus cannot grow through intact glumes and the spores must be brought into the open florets by insects or by aerosols induced by rain and wind. Therefore, cross-pollinating grasses, such as rye, are a primary target of the fungus. The infection success of the fungus is reduced when the florets are already pollinated and the then-closed glumes prevent access to the stigma. Ergot spores mimic the fertilization process of rye [8]. After landing on the stigma, the germ tubes grow through the pistil, like the pollen tubes, and infect the ovary exclusively. Therefore, the pollination has a considerable effect on infection frequency and disease development [9]. The combination of less available pollen, poor pollen flight properties, and an extended opening time of the unfertilized florets in rainy weather gives the fungus a competitive advantage in relation to pollen. This is less pronounced as the cultivar sheds more pollen (Figure 1b).

In rye, population varieties consist of self-incompatible plants, whereas hybrid varieties descend from self-fertile parental lines. The different types of varieties have consequences for the possible origin of pollen that is successful in pollinating. Whereas pollen in the first type of variety must bridge the physical distance from one plant to the other, in hybrids that are autogamous (i.e., pollen from the same flower) or geitonogamous (i.e., pollen from another flower but the same plant), selfing is feasible and should, ceteris paribus, lead to an advantage for well-restored hybrids.

The close association between pollen availability and ergot susceptibility poses a problem for hybrid breeding when full pollen shedding cannot be guaranteed due to lack of superior restoration [10]. In the early hybrid cultivars, pollen-fertility restoration was inferior, leading to high ergot contamination when the weather was conducive for the disease. From 1993 to 2004, the mean percentage of natural ergot incidence in the German rye harvest was 0.04% for population cultivars and 0.18% for hybrid cultivars [11]. This was not acceptable, as the EU tolerance limit for ergot sclerotia in commercial grain lots for food at that time was 0.05%, and for feed it was 0.1% [12]. The high susceptibility of the early hybrids to ergot stimulated research for better restorer genes. That story is reviewed here, with special focus on the genetics of restoration, the identification and localization of superior *Rf* genes, and the association between restoration ability and ergot contamination.

## 2. Basics on Cytoplasmic-Male Sterility (CMS) and Restoration of Fertility (*Rf*) in Rye

Hybrid breeding and seed production in rye relies on cytoplasmic-male sterility (CMS) that prevents pollen production, enabling large-scale crossings and corresponding restorer-to-fertility (*Rf*) genes that restore pollen production in the progeny [12]. CMS is a maternally inherited feature in plants, caused by a special cytoplasm leading to an abortion of pollen shortly after the tetrad stage in the meiosis [13]. The cause of CMS is chimeric mitochondrial genes that form open reading frames (*orf*s) that are transcribed and translated to produce a novel protein [14]. They are often co-transcribed with other mitochondrial genes [15], and the resulting protein interacts with mitochondrial respiration. CMS genes are highly species-specific and have been molecular-characterized in several crops, but not in rye. However, it has been found that the presence of extra copies of the mitochondrial genes *cob*, *atpA*, and *atp9* exist in the CMS-inducing Pampa cytoplasm of rye; in the presence of the restorer gene, the transcript level of the additional *cob* gene variants was strongly reduced [16]. The Pampa (P) cytoplasm from an Argentinean landrace is one of several CMS-inducing cytoplasms in rye that gained the most practical importance since its detection [17]. In addition, a second source, the Gülzow (G) cytoplasm from old rye landraces (“Schlägler Alt” and “Norddeutscher Champagner” [18,19,20]) was eventually used for hybrid breeding, but with less impact [21]. Other CMS-inducing cytoplasms are the R-type originating from a Russian population [22,23] and the C-type obtained by crossing the old Polish rye population “Smolickie” with the wild rye *Secale strictum* (syn. *S. montanum*) [24,25]. Other CMS sources not utilized until now were detected in a Swedish inbred line and in several Iranian primitive rye populations collected in the 1950s [26]. Often, the non-P cytoplasms are included under the term Vavilov (V) cytoplasm, because they show similar phenotypic CMS-restorer interactions that are different from the P cytoplasm [27]. The P cytoplasm has the great advantage that the CMS it causes is environmentally very stable and easy to maintain, because >84% of the European gametes are full maintainer genotypes [28]. In a period of two years across five European locations, no pollen fertility occurred among three maintainer lines and their single crosses [27].

Restoration of pollen fertility in the presence of CMS is caused by nuclear-encoded *Rf* genes that control mitochondrial gene expression [29]. They work primarily by preventing translation of mitochondrial CMS genes, either by cleavage of their mRNA, alteration of their size, or degradation [14,29]. In rare cases, post-translational processes also play a role, or non-CMS-associated genes in the mitochondria are involved. Restorer genes that are to be used in hybrid breeding should be fully dominant. The first restorer sources for the P cytoplasm were detected soon after the description of the cytoplasm in inbred lines from European sources [30]. However, in that early study, two out of 13 inbred lines were full restorer for the seed parent line L1-P, among them the still superior restorer line L18; therefore, the problem of incomplete restoration was underestimated. In the early hybrid cultivars, however, the restorer index (0–100% according to [26]) for the P cytoplasm was, on average, 43% and the pollen production relative to a population cultivar was 35% [27]. Because rye is a very effective pollen shedder, this was not a problem for seed set and grain yield. Indeed, the early hybrids already had a higher grain yield than the populations cultivars [4], due to heterosis, but they also had a higher ergot incidence.

The main cause for the partial restoration was the inferiority of European restorer sources for the P cytoplasm. The frequency of effective restorer gametes in European rye populations is low (3–4%); restoration is highly dependent on the environment and the seed parent genotype, often leading only to incomplete restoration [27]. Moreover, European restorer sources have an oligogenic inheritance of pollen-fertility restoration. Often a major gene with a large effect is complemented by several minor genes. For example, the old inbred line L18, which to this day is among the best European restorer lines, had a major *Rf* gene on chromosome 1RS, with 54% of explained phenotypic variance, but leading only to a restorer index of 55%. This locus was already detected by an isozyme marker [31]. Additionally, minor *Rf* genes on chromosomes 3RL, 4RL, and 5R, with 9% to 17% of explained phenotypic variance, were found [32]. A second European line, L161, showed two major *Rf* loci on chromosomes 1RS and 6R [33,34]. This mode of inheritance coincides with the study of Scoles and Evans [35] that postulated three dominant genes for restoration of the P cytoplasm, with high environmental effects. They already mentioned that oligogenic inheritance leads to the occurrence of a high percentage of partially male sterile plants, a feature that was observed in all studies of pollen-fertility restoration [21,30,32]. They are useful for developing neither maintainer nor restorer lines, but they disturb the selection of high pollen-fertility restoration because they are highly prone to environmental variation. This is caused by complex interactions between *Rf* gene expression and temperature and photoperiod conditions [14]. Thus, the relative pollen amount in P cytoplasm might range, for the same restored single cross, from 22% to 70%, depending on the location-year combination [28].

## 3. Detection, Distribution, and Effects of *Rf* Genes from Genetic Resources

The inferiority of European sources for restoring CMS derived from P cytoplasm led to the search for new restorer sources. In 1990, at the University of Hohenheim, we crossed a CMS single cross that was hard to restore with non-adapted rye populations, and in the following year we visually analyzed the progenies with 120–160 single plants to assess their pollen fertility restoration. While four European populations and one Canadian population that were used as standards had only restorer frequencies from 2% to 6%, in contrast the non-adapted populations showed restorer frequencies of 12% to 58% [34]. They were derived either from Argentinean landraces (“Don Enrique”, “Pico Gentario”, “Pastoreo Massaux”, “Insave”, “Manfredi”, “Safico”, “Trenelense”) or from Iranian weedy rye (“Altevogt 14158–14162”, “IRAN I”, “IRAN III”, “IRAN IX”) that are similar to those from which the original P cytoplasm was extracted or, in one case, from Turkish weedy rye collected in wheat fields there in the 1990s. Interestingly, in some of these accessions, CMS sources were described earlier [26]; among them were four single plants from an Argentinean landrace and ten accessions from the Iranian primitive ryes originally collected by Kuckuck [36] and later named from “IRAN I” to “IRAN IX’. Subsequently, Stojałowski et al. [37] detected several single plants with a specific marker, with P cytoplasm in five of the Argentinean landraces and in two of the Iranian primitive rye populations (“IRAN I” and “IRAN IX”) mentioned above. In three “Altevogt” accessions, however, their marker assay did not find any plant with P cytoplasm, although we detected *Rfp3* in one of these accessions. The difference between our phenotypic tests with CMS materials and the marker analyses of populations is that the pollen-fertile progenies can be easily filtered out visually during flowering from hundreds of plants growing in the field, while the population size analyzed by markers is restricted. Obviously, a coevolution between the spontaneous emergence of a CMS-inducing cytoplasm and the development of *Rf* genes has occurred in rye, as expected based on theory [14]. Indeed, populations containing high proportions of CMS plants can only survive over the long term if they also possess *Rf* genes, especially if the population size is limited [29], as is often the case in populations of wild plants.

**Figure 2:**
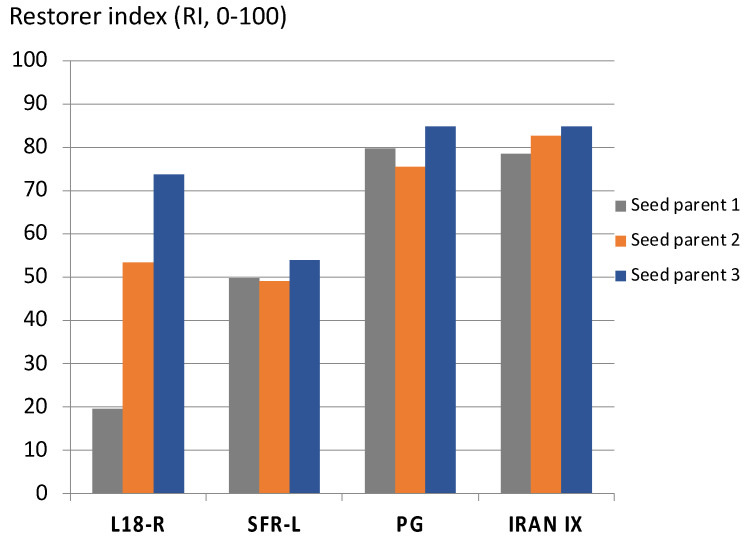
Restorer indices (0–100%) of factorial crosses between four pollinator and three CMS-seed parent lines in P cytoplasm across 3 locations (PG = Pico Gentario; Data source: [38]).

The new restorer genes proved to be much more effective than the European sources, and less dependent on the seed parent (Figure 2). In this comparison, L18-R is the worst restorer line, although it is one of the best European sources. SFR-L resulted from several cycles of a recurrent selection scheme for pollen-fertility restoration, illustrating that improvement is also possible within European sources. The two non-adapted sources, however, had the highest restorer indices of about 80% and were able to restore all three seed parents to the same amount. The results of L18-R clearly show that the seed parents were, in different ways, hard to restore. Specific combining ability (SCA) effects cannot be seen in SFR-L, PG, and IRAN IX [38]. Moreover, the non-adapted *Rf* genes were able to restore a near-isogenic line in six other CMS-inducing cytoplasms of differing origin [38], revealing the characteristics of a “universal restorer”.

**Figure 3:**
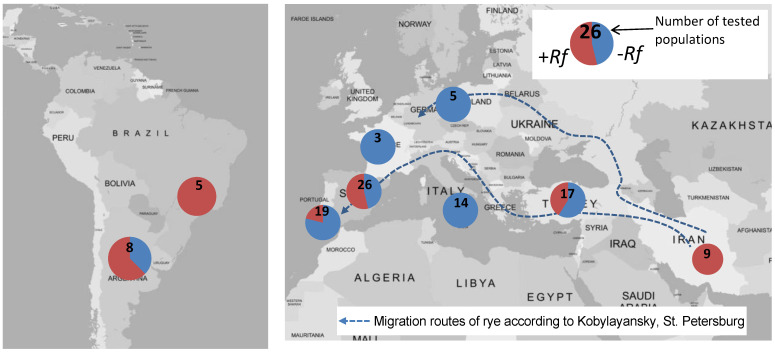
Frequency of *Rfp1* from IRAN IX among 106 rye populations as tested by a gene-specific molecular marker.

Interestingly, the non-adapted *Rf* genes are not equally distributed geographically among rye populations, as shown for the *Rfp1* gene from IRAN IX (Figure 3). A survey of 106 rye populations with a DNA marker very closely linked to *Rfp1* showed a 100% frequency of this gene in nine Iranian primitive rye populations, one of which it was derived from, and high frequencies in Turkish weedy rye, Spanish and Portuguese populations, and Brazilian and Argentinean populations. It was never found in German, French, or Italian populations, or in Russian populations [39]. This indicates that the *Rfp1* gene was not transported on the routes that are usually discussed for rye migration during past decades [1,40]. The main question is how *Rfp1* came from its origin in southwestern Asia to Spain and Portugal, and finally to South America, without touching the main areas of rye-growing in central and eastern Europe. The neolithization of the Iberian Peninsula is very similar to what we know from other neolithic settlements in Europe, accompanied by the classical “founder crop” package with einkorn, emmer, and barley as cereals, but not rye [40]. However, there are many clues from the centuries that followed that distinguish the peninsula’s past from that of the rest of Europe. According to whole-genome data analysis, the Iberian rye material clustered together with wild material or with domesticated or weedy rye accessions from the Levant, and it is clearly distinct from northern and central European samples [41]. Whether Phoenician traders (around 1100 BCE, before common era) or Moorish (~200 CE, common era) groups brought the grain into these countries has not been clear until now [40]. Obviously, the *Rfp1* gene follows this pattern, showing the distinctive role of Iberia.

## 4. Introgression of *Rf* Genes into Modern Cultivars, a Unique Success Story of Markers

**Figure 4:**
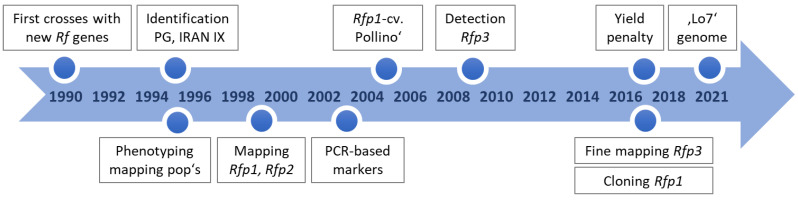
Time course of detection and phenotypic and molecular analyses of effective, non-adapted restorer genes.

Soon after the detection of highly effective *Rf* genes in Iranian and Argentinean rye populations, mapping projects started to analyze the genetic architecture of this trait and to reveal molecular markers for a targeted introgression into elite material (Figure 4). While the two mapped European restorer lines L18 and L161 clearly had an oligogenic inheritance, the non-adapted populations showed a preponderance of one major locus on chromosome 4RL, explaining 68% and 59% of the phenotypic variation for IRAN IX and Pico Gentario, respectively [32]. The loci were named *Rfp1* and *Rfp2*, respectively. A third locus, *Rfp3*, was contributed by the Iranian primitive rye Altevogt 14160 [42] and fine-mapped to the same genomic segment [43].

The first mappings were done with RFLP and AFLP markers that are not convenient for routine marker-assisted backcrossing. Development of sequence-specific primers for *Rfp1* and *Rfp2* resulted in estimated marker distances of 1.2 cM for IRAN IX and 2.2 cM for Pico Gentario [44]. When calculating the linkage drag based on a marker bracket that flanked an interval of 2.9 cM or 5.2 cM, respectively, this results in an estimated length of the donor chromosome segment that is, on average, 45 cM in BC4 and 32 cM in BC6 (rf. to [45]). This illustrates that linked inferior donor genes can easily be introduced into the restorer gene pool during marker-assisted backcrossing. This can only be reduced when maximal recombination is allowed during the backcrossing to break up such linkages.

Stojalowski et al. [46] used these markers and mapped them in the vicinity of the *Rfc1* that restores pollen fertility of the C cytoplasm, implying a close relationship of the corresponding *Rf* genes.

Fine mapping of the *Rfp1* gene with >4500 plants revealed a marker only 0.093 cM apart from the gene [47]. Meanwhile, a gene-derived marker is used for backcrossing purposes and the company KWS LOCHOW GMBH has introduced the brand “PollenPlus^®^” [48] to designate their commercial hybrid cultivars that include *Rfp1.*

## 5. Non-Adapted *Rf* Genes Show Lower Grain Yield and Taller Progeny

Already in the first mapping study, *Rfp2* from Pico Gentario showed a close linkage to a QTL for plant height that, on average, made the plants possessing *Rfp2* 13.4 cm taller [32]. To further analyze possible side effects of non-adapted *Rfp* genes, we used BC4S2 backcross lines tracing back to a BCxS1 single plant as last common ancestor with and without *Rfp3* from Altevogt 14160 [49]. The chromosome segment carrying *Rfp3* could be delimited to a 2.5 cM interval by newly derived COS (conserved orthologous gene sets) markers on chromosome 4RL [50]. Accordingly, we developed near-isogenic BC4 and BC5 lines, with and without an *Rf* gene from an Iranian primitive rye (IRAN III), from another Argentinean population (“Trenelense”) and the *Rfp2* gene from Pico Gentario, and tested them in six environments by crossing to a single cross that was difficult to restore (Table 1).

The restorer index of the lines with the respective *Rf* gene was significantly higher than that of the lines without *Rf* genes, again illustrating the high effectiveness of the non-adapted restorer genes. However, plants with *Rfp* genes are, on average, significantly lower yielding and taller. This yield penalty can only partially be attributed to reduced thousand-kernel weight; it is also likely affected by other yield components. The yield penalty appears all the greater, considering that interpool three-way hybrids showing full heterosis and plot yields >82 dt/ha were tested here. However, the yield penalty ranged among seven comparisons from Altevogt 14160, for example, from −0.95 dt/ha to −10.01 dt/ha, showing that there are possibilities for improvement. The possible causes for the lower yielding and taller plants that are restored by an effective *Rfp* gene are manifold: linkage with unfavorable genes from the non-adapted donor, pleiotropy due to the *Rf* gene action (“costs of restoration”), or epistatic interactions of the *Rfp* gene with other genes of the receiving parent. Interestingly, all non-adapted sources of *Rfp* genes showed these unwanted effects, irrespective of whether they were derived from Iranian primitive rye or Argentinean landraces. This points to a systemic effect of the *Rfp* genes. Linkage drag is also less probable, because even much higher backcross generations than those analyzed here show yield penalty [51]. Moreover, the restorer lines with *Rfp1* still have a clearly noticeable altered phenotype. They are earlier in heading, taller with a higher lodging susceptibility, and have a higher ear glaucousness and hairiness of the culm and a lower grain weight [51]. This strengthens the evidence that these effects are pleiotropic in nature and are directly due to the *Rfp1* gene.

Negative pleiotropic effects, i.e., the fitness costs of restoration, were broadly discussed among botanists for natural plant populations, because in gynodioecious populations, i.e., where hermaphrodites and male-sterile plants are coexisting, *Rf* genes should approach fixation when no costs are considered [29], although that has never been observed. From a theoretical point of view, the pleiotropic costs of restoration should occur when (1) the costs are dominant (costs that are independent from *Rf* gene action) and constitutive (costs that occur regardless of the CMS background); (2) the *Rf* genes interact with transcripts not produced by the CMS gene; and (3) the tissue specifity of the *Rf* genes is low or absent, affecting pollen and seed production [29]. In this case, the restorer locus in a natural plant population only rarely occurs, as has been found in rye. The *Rf1* gene restoring the CMS-T cytoplasm in maize, for example, is very rare and is expressed in multiple tissues affecting seed and pollen production. However, the effect of *Rf1* is specific to the CMS-related transcripts and should be not constitutive. Duvick [52] reported previously that restored plants from the T cytoplasm were on average 10.1 cm taller than non-restored plants and have a significant yield penalty, although the penalty is much lower than reported from rye. However, it is not clear which restorer genes used by Duvick corresponded to the modern nomenclature. *Rf3* restoring the S cytoplasm of maize, for example, has a pleiotropic effect, not only on the chimeric CMS gene but also on two other essential mitochondrial genes, *cob* and *atp6* [29]. This effect is dominant and observed in all tissues. In rapeseed (*Brassica napus*), Montgomery et al. [53] found significant differences between near-isogenic male-sterile and restored hybrids, but only for flowering traits, not for yield-related traits. Interestingly, in that paper, the costs of restoration differed according to the tested CMS/*Rf* gene combination. No costs occurred for *Rfp* restoring the pol cytoplasm, while *Rfn* restoring the nap cytoplasm had pleiotropic effects, reducing flower size, stamen length, and pollen counts. Because European *Rf* genes in rye show only partial restoration, at best the costs of restoration might have such small effects that they do not become significant and occur only with the non-adapted *Rf* genes. Another possibility that could additionally or alternatively apply is that male-sterile plants may not need to invest resources in energy-consuming pollen production, and therefore may realize higher grain yields when non-restored, as previously shown in maize [54]. In hybrid breeding programs, restorer lines are usually validated by testcrosses formed by outcrossing the lines on CMS testers from the seed parent pool. Accordingly, candidate lines lacking effective restorer genes benefit from a yield advantage and have a higher chance of being recombined for the next breeding cycle. Accumulated over several breeding cycles, this reduces the frequency of restorer genes in the advanced breeding population.

Whatever the reason for the reduced yield and increased plant height, hybrid rye breeders must find a solution if the superior non-adapted *Rf* genes should be used for reducing ergot susceptibility in commercial hybrid cultivars.

## 6. Genomics of Rye *Rf* Genes—Synteny and Structural Organisation of *Rf* Loci

*Rfp1* and *Rfp2* were both located on chromosome 4RL (Table 2). In Altevogt 14160, another major locus on chromosome 4RL was detected, *Rfp3*, as well as a minor *Rf* gene on chromosome 1R [42]. The three major *Rfp* genes were located by gene-derived conserved ortholog set (COS) markers in the same small orthologous segment on chromosome 4RL [43]. A comprehensive literature review showed that *Rf* genes of different rye sources and cytoplasms, from different *Triticeae* and maize, are located in the syntenic segment of chromosome 4RL (Table 2). Most prominent, the barley restorer gene *Rfm1* resides in the same syntenic 4RL/6HS segment [43], and an RFLP probe of *Rf2* from maize could be assigned to the homoelogous rye chromosome 4RL in the L18 background [55]. This supports the assumption that the three *Rfp* genes in this segment either belong to a family of closely linked genes, or are allelic. In a previous study, Miedaner et al. [32] reported that they did not find any segregation in a population of 2060 single plants derived from a cross between two inbred lines possessing either *Rfp1* or *Rfp2*, where all progenies were fully male fertile.

Interestingly, in the same subgenomic region, *Rfg1*, a major *Rf* gene for the G cytoplasm, was mapped [56], as well as the rye *Rf* gene *Rfc1* interacting with the C cytoplasm [57] and a further rye-derived *Rfc4* gene restoring the CMS-inducing *Triticum timopheevi* cytoplasm in hexaploid wheat. Moreover, two wheat loci restoring the same cytoplasm, *Rf4* and *Rf6*, are located on the syntenic 4RL/6AS and 6BS segment [58,59]. So, major *Rf* genes of six different CMS systems have been allocated to this segment.

The whole-genome assembly of inbred line “Lo6” found in this 4RL segment a cluster of mTERFs (mitochondrial transcription termination factor) and PPRs (pentatricopeptide repeat) that are classified as *Rf like* (RFL, [65]). In total, 83 RFL-PPRs and 131 mTerfs were annotated in “Lo6”. Rabanus-Wallace at al. [65] found that *Rfp1, Rfp2,* and *Rfp3* on the one hand, and *Rfc1* on the other, are closely linked but physically distinct. Two members of the RFL-PPR clade reside within 0.186 Mb of the *Rfc1* locus. These data for chromosome 4RL agree with a postulated “hotspot of mito-nuclear interaction” [66].

**Figure 5:**
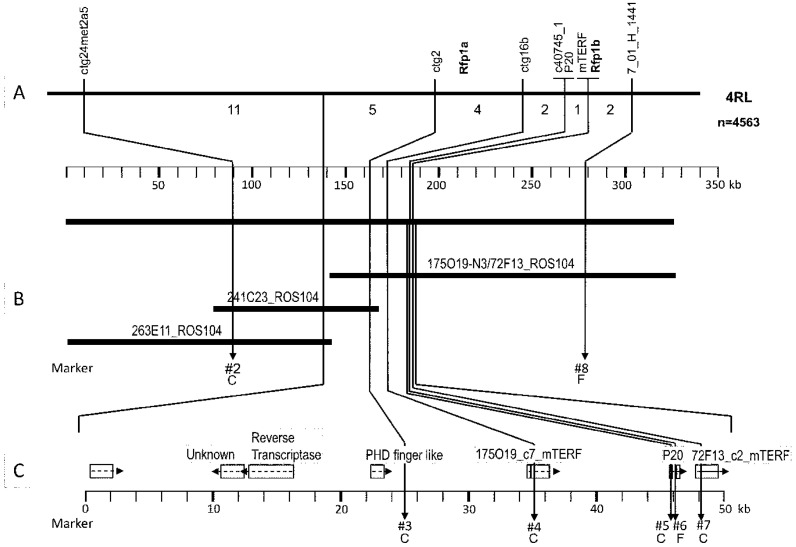
Organization of the *Rfp1* locus on chromosome 4RL [47]. (**A**). number of recombinations between named markers; (**B**). overlapping BAC clones; (**C**). putative candidate genes.

The *Rfp* loci, in contrast, are neighbored by four *mTERF* genes [65], in agreement with a previous report that a mTERF protein represents a *Rfp1* candidate gene [47]. It could be shown for the first time that pollen fertility restoration in this introgression segment from IRAN IX is caused by two independent but tightly linked and almost equivalently acting *Rf* genes (*Rfp1a*, *Rfp1b*, Figure 5).

The *Rfp1b* gene encodes an mTERF protein and *Rfp1a* also shows very high concordance with another *mTERF* gene. Similarly, *mTERF* genes were closely linked to *Rfm3* on barley chromosome 6HS [63] and to wheat *Rf9* on the 6BS chromosome [67], both regions syntenic to rye 4RL. A major *Rf* gene for the G cytoplasm on chromosome 3RL also belongs to the *mTERF* gene family [21], although this locus seems to be distinct from the known P- and C-type systems in rye and the CMS systems in barley and wheat. Generally, *mTERF* genes encode helical repeat proteins that target mitochondrial DNA, thus suppressing the expression of mitochondrial CMS genes that lead to male sterility [68].

*Rfp1a* and *Rfp1b* also individually lead to complete restoration in most cases. However, the effects on grain yield differed, depending on how many loci were available [47]. If both loci carried the donor allele of IRAN IX, the yield loss was about twice as high as when only the donor allele of *Rfp1b* was present (7.0 vs. 3.7 dt/ha). In recombinants carrying only the donor allele of *Rfp1a*, the yield loss ranged from 3.7 to 4.9 dt/ha. Thus, a very tightly linked marker could be obtained in this study, as well as a recombinant plant with a yield loss reduced by half.

On chromosome 1RS *Rf* genes were repeatedly found in diverse genetic rye materials, as a major locus in L18, as minor loci in Pico Massaux and Altevogt 14160, and, more recently, as an *Rf* locus for the G cytoplasm [21]. This might coincide with the wheat locus *Rf^multi^* (*restoration-of-fertility in multiple CMS systems*) on wheat chromosome 1BS. Here, wheat and rye share a RFL-PPR gene cluster [65]. Only two wheat RFL-PPR genes encoding full length proteins reside in the cluster; one of them corresponds to a putative rye ortholog. The absence of the other ortholog in the sequenced non-restorer genotype of rye “Lo7” suggests it as an attractive candidate gene for the *Rf^multi^* locus [65]. This region on 1RS harbors 22 out of the 83 annotated RFL-PPR genes in the “Lo7” reference genome [65]. In addition, the RFL-PPR genes *Rf1* and *Rf3* restoring the wheat *Timopheevi* cytoplasm reside on this syntenic chromosome. They were shown to bind to a mitochondrial *orf279* transcript, to induce cleavage, and thus to prevent the expression of the sterilizing factor [69].

## 7. Superior *Rf* Hybrids Show Considerably Less Ergot

**Figure 6:**
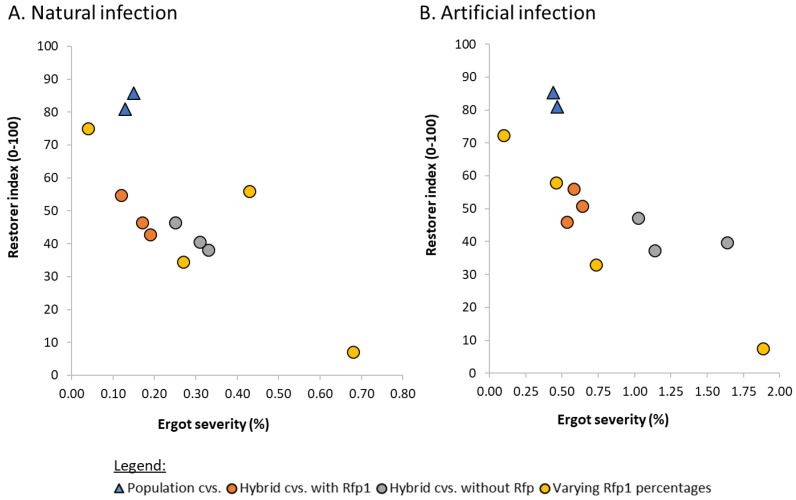
Ergot severity and restorer index of different types of cultivars (cvs., varying Rfp1 percentages are 100%, 50%, 25%, 0%, respectively) with (**A**) and (**B**), both across eight locations (Data source: (**A**) unpublished, (**B**) [71]).

The association between pollen shedding ability and contamination by ergot has been established in several studies [10,70,71]. In particular, when European *Rf* genes are used that are not as effective as non-adapted *Rf* genes, the higher ergot susceptibility becomes obvious. This is more pronounced in artificial infections because disease pressure is higher, but it can also be seen in natural infections in a similar way (Figure 6). Single-cross hybrids with 100% *Rfp1* have the highest pollen production level, reaching population cultivars and an even lower ergot severity. Reducing the percentage of the *Rfp1* gene to 50% clearly results in less pollen and a higher ergot severity. Commercial hybrids with *Rfp1* are in the same range for both traits. Hybrids without these *Rfp* genes are clearly inferior in restorer index and ergot severity, which is particularly evident under artificial infection. A single cross without any *Rfp* gene marks the maximal ergot susceptibility. The correlation between natural and artificial infections was very high (r = 0.94; *p* ≤ 0.001). In conclusion, the integration of non-adapted *Rfp* genes can result in hybrids that have a low ergot severity similar to that of population cultivars.

**Figure 7:**
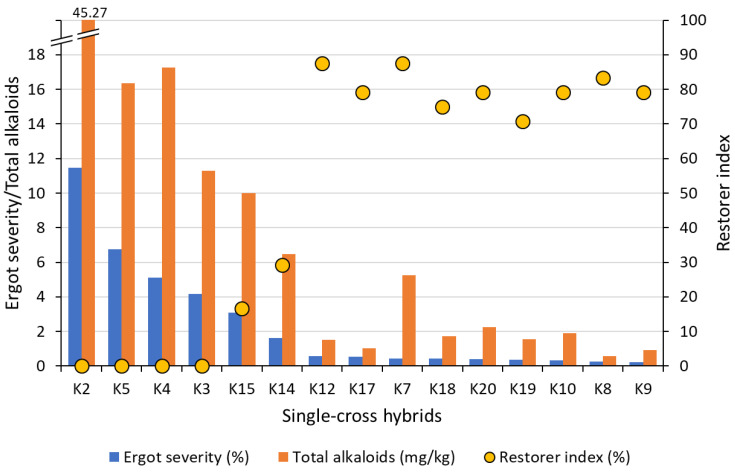
Association between ergot severity, total alkaloid content, and restorer index for 15 single-cross hybrids with and without *Rfp1* across two location-year combinations (Data source: [10]).

Because the new EU limits for ergot regulate total alkaloid content (see next Section), reduced ergot susceptibility alone is not enough; the alkaloid content in the harvested grain must also be low. A recent experiment with 15 single-cross hybrids in a very wide range in the restorer index, from 0–90%, showed a high correlation between reduced ergot susceptibility and alkaloid content (r = 0.98, *p* ≤ 0.001; Figure 7). Because ergot susceptibility is directly associated with a low restorer index (r = –0.838, *p* ≤ 0.001; Figure 7), selection for high pollen-fertility restoration might already suffice to meet the stricter alkaloid regulations.

## 8. How to Proceed in Future Breeding

Hybrid breeding is the future of rye! The higher yield performance allows the crop to compete with wheat, which usually fetches a higher price on the market. In view of climate change, with drier and warmer conditions in the future, hybrid rye is superior in all growing regions. It also requires less pesticide and nitrogen fertilization, in line with the European Green Deal, which calls for higher environmental protection standards in agriculture. The EU has significantly tightened the limits for ergot since 1 January 2022. In addition to the previous limit of a maximum of 0.5 g/kg sclerotia and sclerotial fragments on unprocessed rye, harvested grain may not contain more than 500 µg/kg total alkaloids on rye milling products for human consumption [72]. From 1 July 2024, the limits will be reduced to 0.2 g/kg sclerotia and a maximum of 250 µg/kg alkaloids. Therefore, the high pollen-shedding of hybrid varieties must be the top priority to avoid ergot.

**Figure 8:**
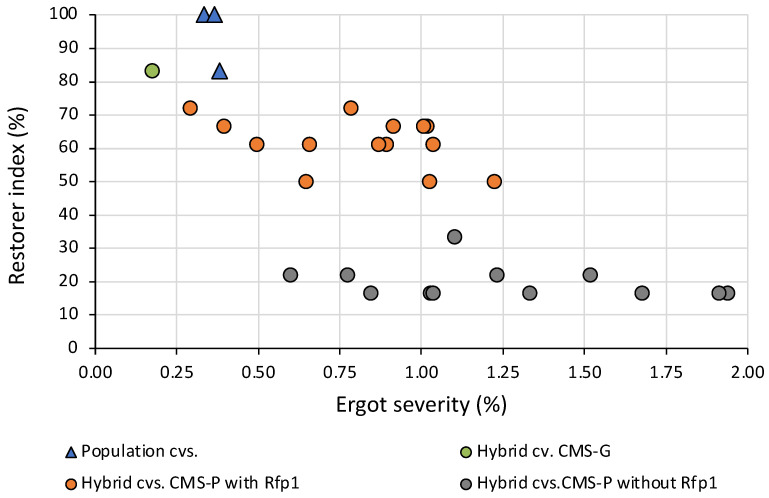
Ergot severity and restorer index of the entries of the Official VCU trials from Germany in 2021 after artificial ergot infection across four locations (cv./cvs = cultivar/s, G=G cytoplasm, P=P cytoplasm).

Besides maximizing pollen-shedding by using non-adapted *Rfp* genes, the question of resistance of rye to the pathogen, *Claviceps purpurea*, arises. It was previously taken for granted that ergot infection does not cause any defense reactions in rye. However, recent transcriptomic studies showed that in both wheat and rye, the host plant responds to the infection by upregulating defense-related genes, precisely when the germ tube reaches the ovary [73,74]. Thus, the host obviously recognizes the *Claviceps* infection. Indeed, it has been shown several times that diverse sets of both fully pollen-fertile rye and fully male-sterile rye, when grown under pollen-isolated conditions, showed small but significant differences in their ergot reaction [75,76,77]. These quantitative maternal differences could be exploited by targeted selection, as shown by some commercial hybrids without *Rfp1* genes and with a low restorer index, as well as lower ergot severity even after artificial infection (Figure 8, please refer also to Figure S1). In Germany, Poland, and Austria, the official cultivar registration procedure includes multi-locational testing for ergot severity over two years, with artificial inoculation in a special experimental setting [70].

Alternatively, a lower ergot severity without non-adapted *Rfp* genes could be reached using seed parent lines that are easier to restore, in combination with weaker European restorer genes that have no yield penalty. The resulting hybrids will shed more pollen and might, under favorable conditions, even reach the effect of the non-adapted *Rf* genes (rf. to Figure 2, seed parent 3). However, it must be ensured that they also inherit the non-restorer trait in an equally stable manner. The existence of modifier genes in the seed-parent pool that are effective only in presence of a major *Rf* gene and that enhance its expression has already been demonstrated on chromosome 6R of inbred line Lo 7 [32]. In concrete cases, the decision of the breeder regarding how to build up a hybrid might depend on the availability of CMS single crosses that are easy to restore, and on the presence of minor restorer genes in the restorer synthetic.

Still, the high effectiveness of non-adapted *Rf* genes will play a prominent role in future hybrid breeding. The main task is to reduce the obvious yield penalty and other negative agronomic features. Even if the introgressed genomic section is significantly shortened and restricted to one of the two *Rfp1* loci, a significant yield loss remains, even in three-way hybrids [47] that could not be further reduced using more closely linked markers. However, the effects of the same *Rfp* gene on different genotypes significantly differs, as shown above; therefore, a consequent selection in the elite gene pools for mitigating undesired side effects of *Rfp1* genes seems feasible in the long run. This would replicate the coevolution that has occurred in adapted CMS/*Rf* systems over evolutionary time periods [14].

This coevolution could be enhanced in a breeding context, when environmentally stable adaptation genes exist in the elite gene pools that lower the negative agronomic effects of non-adapted *Rfp* genes. Research activities should be begun to identify and to exploit these genes. In the pollen parent population, elite lines already carrying the *Rfp* gene could be crossed with non-carrier lines to make sure that most of the resulting F1-individuals are heterozygous for the *Rfp* gene. From the subsequent selfing process, recombinant inbred lines, e.g., in S2 generation, will appear, which will be homozygous *Rfp* carriers and non-carriers. Following the regular breeding scheme [12], these two groups of restorer lines, tracing back to the same parental lines, can be outcrossed to testers from the seed parent pool and tested for their breeding value to this pool. In a subsequent GWAS, marker effects in these two groups should be similar for all QTL that do not depend on *Rfp* presence/absence. Conversely, marker effects should differ for adaptation QTL transmitting a lower or higher yield penalty from the *Rfp* gene. These differences can be used to identify candidate QTL in the pollen parent gene pool relevant for adaptation to the presence of the *Rfp* gene. Further, the respective genomic information can be used to calibrate the formula for predicting breeding values of selection candidates when the *Rfp* gene is present. An analogous procedure can be pursued in the seed parent pool. Here, CMS-versions of inbred lines can be top-crossed to near-isogenic pollen parent testers that are fixed either for *Rfp* presence or absence. When comparing the two tester groups in a subsequent GWAS, their marker effects should differ for QTL controlling adaptation to *Rfp*, and the prediction formula can be adjusted to maximize the correlation to the target criterion, which is the breeding value in presence of *Rfp*. Testers should be near isogenic to avoid confounding specific combining and adaptation gene effects. In a modern breeding program, candidate inbred lines will be subjected to genomic selection anyway [12]; therefore, these research activities would cause little extra cost and allow work with large population sizes. Across several cycles, the yield penalty should be reduced with the increasing frequencies of positive adaptation alleles.

As pointed out in Section 4, the *Rfp* gene could be introgressed into the pollen parent pool by backcrossing the highest performing inbred lines carrying the *Rfp* gene as donors to more recent elite lines as recipient parents. By selecting on *Rfp* presence, a selective sweep will be generated, resulting in a large reduction of genetic variation in the adjacent chromosome region. Marker-assisted background selection (MABS) [78] can be applied to reduce the donor genome attached to the target gene. However, MABS is costly and often needs a multi-generation approach, with many backcross individuals to be screened for single or double recombination events in the respective region. By organizing crosses in the pollen parent pool, as described above, most of the resulting F1-individuals will be heterozygous for the *Rfp* gene. Thus, the chances for recombination events in the vicinity of the *Rfp* gene are high, allowing the recovery of genetic variation in such a region. There is little doubt that in the long run the highest mitigation effect on ergot can be achieved if the pollen parent pool is fixed for the *Rfp* gene and, accordingly, *p*(*Rfp*) = 1.0 in all restorer synthetics used as male parents of commercial hybrids. Nevertheless, in a transition phase used to accumulate the adaptation genes discussed above, restorer allele frequencies 0.5 < *p*(*Rfp*) < 0.75 could be a meaningful compromise in balancing yield penalty and ergot susceptibility.

Another breeding scenario could be the use of alternative CMS-inducing cytoplasms, of which the G cytoplasm is the best developed at present [21]. There are existing non-restorer and restorer lines; the pollen-fertility restoration is generally much higher than with CMS-P, and the ergot contamination is lower, as can be seen from a VCU-tested commercial hybrid (Figure 8). In this case, an intensive marker-assisted enrichment of reliable non-restorer genes in the seed parent pool must be initiated.

Considering that the P cytoplasm is known to be very stable environmentally, and elite pools are adapted to this cytoplasm, it is recommended that it should be used further, and that elite gene pools be adapted to the non-adapted *Rfp* genes. In conclusion, the development of commercial hybrid cultivars in P cytoplasm, with a similarly low or an even lower ergot severity than population cultivars, appears to be promising.

## Figures and Tables

**Table 1:** *Rf* effect (=difference between backcross lines with and without *Rf* gene) for restorer index (RI), plant height (PH), grain yield (GY), and thousand-kernel weight (TKW); *n* = number of independent comparisons.

Population	*n*	RI (%)	PH (cm)	GY (dt/ha)	TKW (g)
Altevogt 14160	7	+80.2 ***	+9.3 **	−4.1 **	−0.88
IRAN III	9	+51.0 ***	+3.7 *	−8.4 **	−1.30
Trenelense	3	+47.3 ***	+4.5 *	−5.7	−1.01
Pico Gentario	5	+52.7 ***	+7.2 ***	−9.1 **	−1.99 *

*, **, *** Significant at *p* = 0.05, 0.01, and 0.001, respectively.

**Table 2:** Restorer genes mapped in syntenic regions of rye, wheat, barley, and maize (minor genes in brackets).

Species	Restorer Gene	Cytoplasm ^a^	Homoelogous Chromosome Region in Rye					Reference
			1R	3R	4RL	5R	6R	
Rye	L18-R	P	1RS	(3RL)	(4RL)	(5RL)		[32]
	L161-R	P	1RS				6RL	[33]
	*Rfp1*	P			4RL			[32]
	*Rfp2*	P			4RL			[32]
	*Rfp3*	P	(1R)		4RL			[42,43]
	*Rfp_PM_*	P	(1R)		4RL			[60]
	*Rfg2, Rfg1, Rfg3*	G		(3R)	4RL		(6RL)	[19,56]
	*RfNOS1*	G	(1RS)	3RL				[21]
	*Rfc1, Rfc2*	C			4RL		6RS	[61]
Wheat-Rye-	*Rcf4, Rcf3*	TIM			4RL		(6RL)	[58]
Add.lines								
Wheat	*Rf1* *Rf3*	TIM TIM	1A 1BS					[62]
	*Rf4* *Rf9*	TIM TIM			6AS 6BS			[59,62]
	*Rf6*	TIM				(5AL)		[62]
Barley	*Rfm1, Rfm3*	msm1,3			6HS			[43,63]
Maize	*Rf2*	T			9			[55,64]

^a^ Abbreviations of cytoplasms: P = Pampa, G = Gülzow, TIM = *T. timopheevi*, T = Texas.

## Data Availability

Not applicable.

## Supplementary Materials

The following supporting information can be downloaded at: https://www.mdpi.com/2223-7747/11/9/1115/s1, Figure S1: Supporting information on Figure 8 with data for four years (instead of one). Ergot severity and restorer index of the entries of the official VCU trials from Germany from 2019 to 2022 after artificial ergot infection across four to five locations (cv./cvs = cultivar/s), CMS = cytoplasmic-male sterility, P = Pampa cytoplasm, G = Gülzow cytoplasm, *Rfp1* = restorer to fertility gene 1 for P cytoplasm from IRAN IX.
