# Correction: Miedaner et al. Effective Pollen-Fertility Restoration Is the Basis of Hybrid Rye Production and Ergot Mitigation. *Plants* 2022, *11*, 1115

**DOI:** 10.3390/plants12122261

**Published:** 2023-06-09

**Authors:** Thomas Miedaner, Viktor Korzun, Peer Wilde

**Affiliations:** 1 State Plant Breeding Institute, University of Hohenheim, Fruwirthstr. 21, 70799 Stuttgart, Germany; 2 KWS SAAT SE & Co. KGaA, Grimsehlstr. 31, 37555 Einbeck, Germany; viktor.korzun@kws.com; 3 KWS LOCHOW GMBH, Ferdinand-von-Lochow-Str. 5, 29303 Bergen, Germany; peer.wilde@kws.com

In the original publication [1], the severity of ergot taken from the German VCU trials was reported from four locations in 2021, as seen in Figure 8. Due to extensive genotype × environment interaction, particularly concerning the effect of European restorer genes and ergot severity, we then add a Supplementary Materials part to extend the original Figure 8 to four consecutive years (Figure S1).

Figure S1 shows that the population cultivars and one hybrid cultivar in the G cytoplasm have the highest restoration index and, consequently, the lowest ergot severity in all four years. Some of the hybrids with *Rfp1* also reach this high level of pollen fertility restoration, whereas the hybrids without *Rfp1* are always inferior to the best cultivar. Due to the effects of maternal genotype and/or genotype × environment interaction, there is an overlap between the hybrids with and without *Rfp1* in the moderate ergot severity classes. This overlap is particularly strong in 2021, as already shown in the original paper, which occurs to a lesser extent in the other years. This may be due to the different levels of ergot severity achieved despite artificial ergot infection. It should also be noted that the artificial infection considerably increases the differentiation between the genotypes (cf. Figure 6 in the original paper).

In conclusion, in the official German VCU trials, some hybrids with the non-adapted *Rfp1* gene and a hybrid in the G cytoplasm achieve such low ergot severity that hybrids without the *Rfp1* gene could not reach.

And Figure S1 citation has also been added in the second paragraph of Section 8.

The corrected text:

These quantitative maternal differences could be exploited by targeted selection, as shown by some commercial hybrids without *Rfp1* genes and with a low restorer index, as well as lower ergot severity even after artificial infection (Figure 8, please refer also to Figure S1).

Newly added Supplementary Materials part:

**Supplementary Materials:** The following supporting information can be downloaded at: https://www.mdpi.com/article/10.3390/plants11091115/s1, Figure S1: Supporting information on Figure 8 with data for four years (instead of one). Ergot severity and restorer index of the entries of the official VCU trials from Germany from 2019 to 2022 after artificial ergot infection across four to five locations (cv./cvs = cultivar/s), CMS = cytoplasmic-male sterility, P = Pampa cytoplasm, G = Gülzow cytoplasm, *Rfp1* = restorer to fertility gene 1 for P cytoplasm from IRAN IX.

The authors state that the scientific conclusions are unaffected. This correction was approved by the Academic Editor. The original publication has also been updated.
