# Supplementary material for: Effective Pollen-Fertility Restoration Is the Basis of Hybrid Rye Production and Ergot Mitigation
Source: Plants (Basel). 2022 Apr 20;11(9):1115. doi: 10.3390/plants11091115 (PMC9104404; doi:10.3390/plants11091115)
Supplement: Supplementary file 1 [file plants-11-01115-s001.zip › supplementary file.pdf]

plants

MDPI

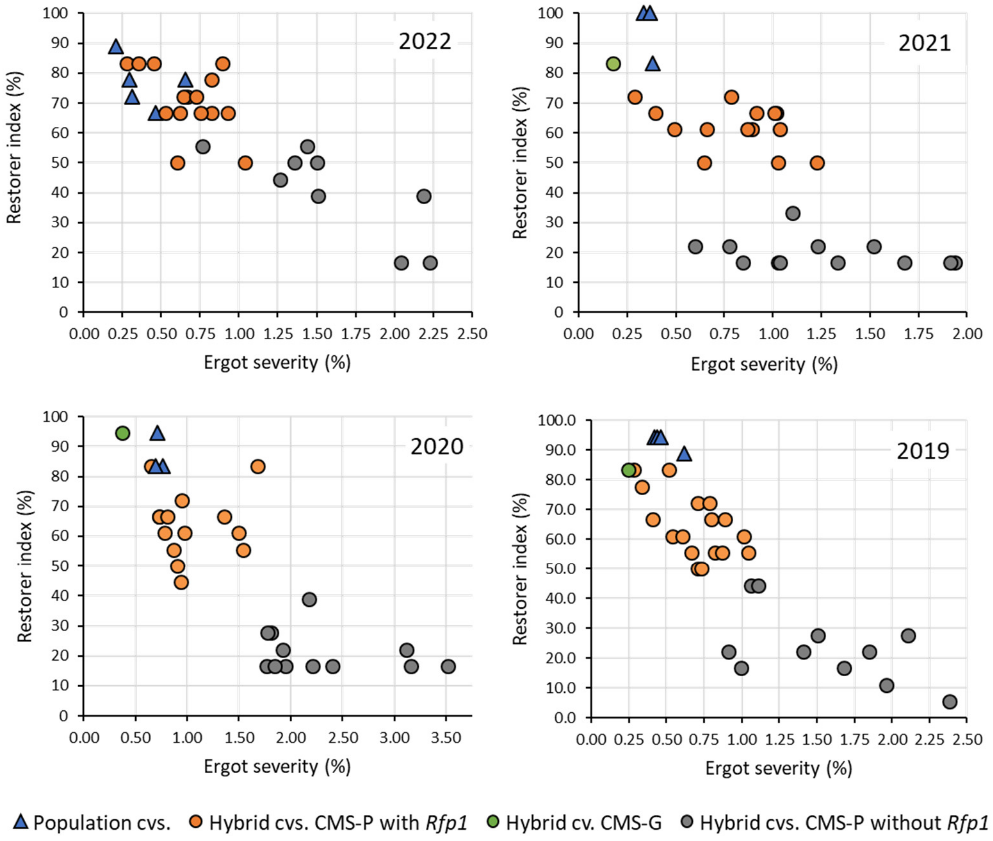

Figure S1. Supporting information on Figure 8 with data for four years (instead of one). Ergot severity and restorer index of the entries of the official VCU trials from Germany from 2019 to 2022 after artificial ergot infection across four to five locations (cv./cvs=cultivar/s), CMS=cytoplasmic-male sterility, P=Pampa cytoplasm, G=Gülzow cytoplasm, *Rfp1*=restorer to fertility gene 1 for P cytoplasm from IRAN IX.
